# Preliminary application of in vivo cardiac diffusion weighted MRI in chronic myocardial infarction porcine model

**DOI:** 10.1186/1532-429X-16-S1-P56

**Published:** 2014-01-16

**Authors:** Christopher T Nguyen, Zhaoyang Fan, Yibin Xie, Behzad Sharif, James Dawkins, Eleni Tseliou, Xiaoming Bi, Rohan Dharmakumar, Eduardo Marbán, Debiao Li

**Affiliations:** 1 Biomedical Imaging Research Institute, Cedars-Sinai Medical Center, Los Angeles, California, USA; 2 Bioengineering, University of California Los Angeles, Los Angeles, California, USA; 3 Cedars-Sinai Heart Institute, Cedars-Sinai Medical Center, Los Angeles, California, USA; 4 Siemens Healthcare, Los Angeles, California, USA

## Background

Cardiac diffusion-weighted MRI is a non-contrast technique that has the potential to identify changes in tissue microstructure in acute myocardial infarction (MI) in humans and rats [1,2]. The trace apparent diffusion coefficient (trADC) was found to be significantly increased in the infarcted region relative to remote regions. The increased trADC is attributed to an increase in extracellular space following cell death, where restriction in water diffusion is less. The aim of this study was to perform in vivo cardiac diffusion-weighted MRI in a chronic MI porcine model to see if this increased trADC chronically persists and correlate it with scar tissue delineated by late gadolinium enhancement (LGE) imaging.

## Methods

Four chronic MI (8 weeks post MI induced by completely occluded proximal LAD) mini pigs were scanned on a 3T MR system (Siemens Verio). Before contrast was administered, M1M2 motion compensated [3] diffusion-prepared 2D segmented TSE was performed to derive trADC maps (TR/TE = 3RR/8.4 ms, α = 180°, 2.1 × 2.1 × 8 mm^3^, 3 slices, diffusion TE_prep _= 105 ms, b = 400 s/mm^2^, G_diff _= 43 mT/m, three orthogonal DW directions). Diffusion preparation was applied in the most quiescent phase identified by CINE imaging (35 phases). Contrast LGE GRE imaging was performed roughly 15 min post contrast injection (TR/TE/TI = 326/1.47/300 ms, α = 20°, 1.3 × 1.3 × 8 mm^3^, 12 slices). The lateral wall of the most basal slice (furthest from the LAD occlusion) was designated as the remote region. The region of infarction was determined by elevated trADC and signal intensity in LGE (both required μ_infarct _>μ_remote _+ 5σ_remote_). For each pig, the mean trADC's calculated for the infarct and remote regions. The infarct area determined by elevated trADC was compared to the LGE infarct area. Differences in means were tested for significance with a two tailed paired t-test. Cardiac phase mismatch between trADC maps and LGE images was corrected using a nonlinear B-spline registration, whose transform was derived from two phases of CINE closest to the trADC map and LGE.

## Results

M1M2 diffusion-prepared TSE scans resulted in mean infarct trADC values of 2.4 ± 0.2 × 10^-3 ^mm^2^/s and mean remote trADC values of 1.4 ± 0.4 × 10^-3 ^mm^2^/s (p < 0.05). The location of elevated trADC infarct region agreed well with the LGE infarct area. The mean trADC and mean LGE infarct area was 0.9 ± 0.1 cm^2 ^and 0.8 ± 0.1 cm^2^, respectively (p = 1). In the infarct regions, wall motion was akinetic.

## Conclusions

In this preliminary study, infarct regions delineated by elevated trADC correlated well with infarct regions defined by protocol matched LGE in area size and location. The roughly 50% increase in trADC values found in the infarct region relative to the remote region was statistically significant. Further histological studies and larger sample size are needed to confirm the origin of the increase in trADC and its relationship with infarcted tissue.

## Funding

NIH/NHLBI RO1 HL38698.

**Figure 1:**
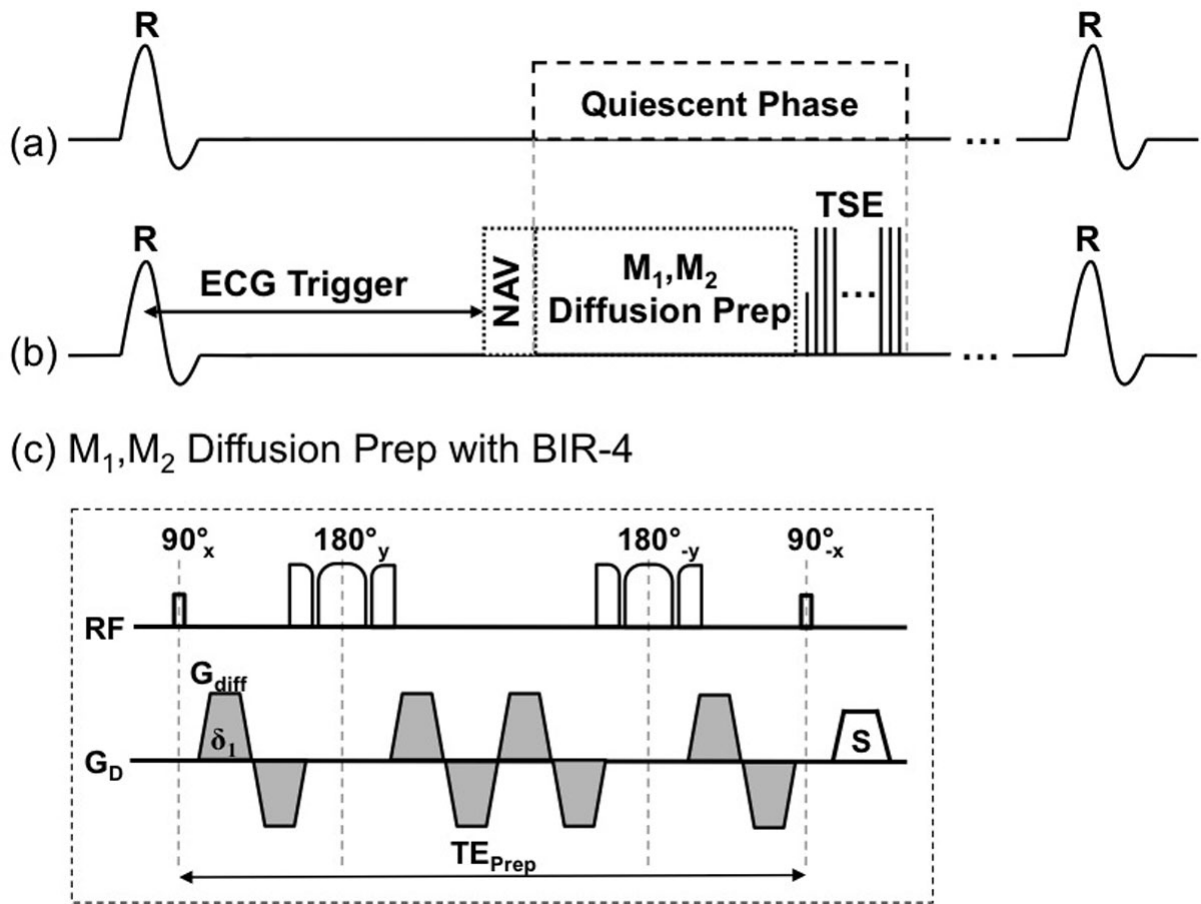
**Pulse sequence diagram**. (a) The longest quiescent period is found using CINE imaging and (b) the motion compensated diffusion prep and segmented TSE readout is placed during this period. In the case the quiescent period is too short to fit both the prep and the readout, the diffusion prep was prioritized and the number of shots were increased. The bulk motion compensated diffusion prep (c) is m1 and m2 compensated and a non-selective adiabatic BIR-4 was used. The magnetization was allowed to recover for 2 RR after the TSE acquisition.

**Figure 2:**
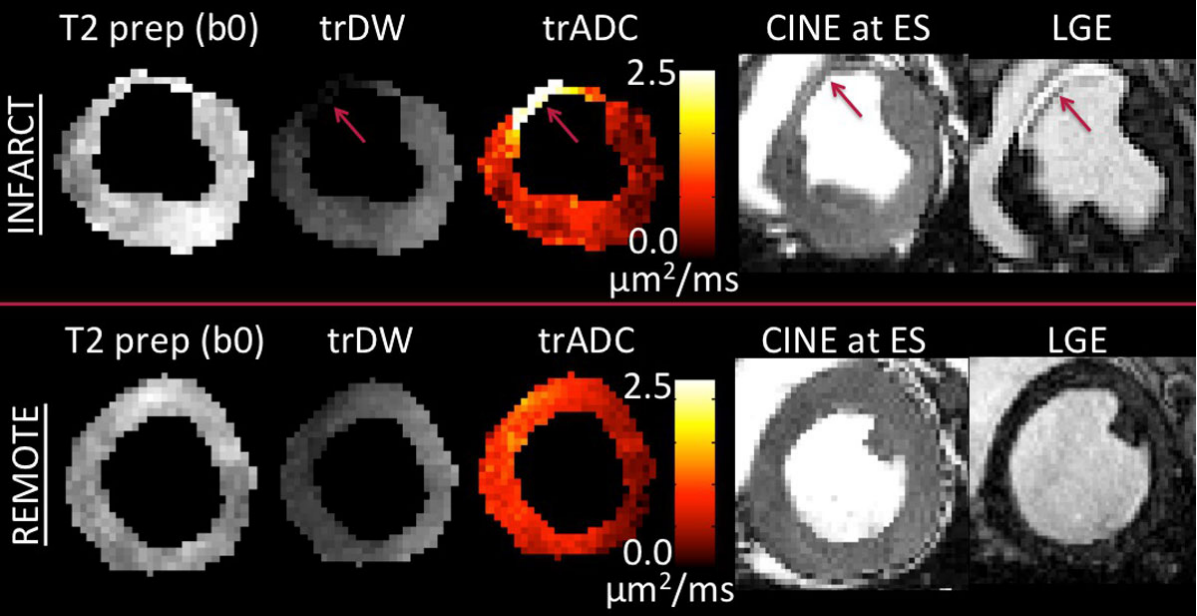
**T2 prep (b0), trace DW, trace ADC, CINE at end systole, and late gadolinium enhanced (LGE) images of the two slices that contained the infarct and remote regions**. Images of the remote regions illustrated no statistically significant changes including the T2 prep image for the infarct slice. In the infarct slice, the trace DW showed a sharp decrease while the trace ADC depicted an increase (red arrows). In the similar region, the LGE image is hyperintense and the CINE image at end systole shows a thin wall that is akinetic. Note the sharp decrease in trace DWI demonstrates that bulk motion corruption is minimal since the myocardium is akinectic in the infarcted region (which would result in a relative increase in trace DWI).
